# Exploring the Role of Apigenin in Neuroinflammation: Insights and Implications

**DOI:** 10.3390/ijms25095041

**Published:** 2024-05-06

**Authors:** Karine Charrière, Vincent Schneider, Manon Perrignon-Sommet, Gérard Lizard, Alexandre Benani, Agnès Jacquin-Piques, Anne Vejux

**Affiliations:** 1 Université de Franche-Comté, CHU Besançon, UMR 1322 LINC, INSERM CIC 1431, 25000 Besançon, France; kcharriere@chu-besancon.fr; 2 Centre des Sciences du Goût et de l’Alimentation, CNRS, INRAE, Institut Agro, Université de Bourgogne, 21000 Dijon, France; vincent.schneider@chu-dijon.fr (V.S.); manon.perrignon-sommet@u-bourgogne.fr (M.P.-S.); alexandre.benani@u-bourgogne.fr (A.B.); agnes.jacquin-piques@chu-dijon.fr (A.J.-P.); 3 Neurology and Clinical Neurophysiology Department, CHU F. Mitterrand, 21000 Dijon, France; 4 Bio-PeroxIL Laboratory, EA7270, University of Bourgogne, 21000 Dijon, France; gerard.lizard@u-bourgogne.fr; 5 Memory Resource and Research Center (CMRR), CHU F. Mitterrand, 21000 Dijon, France

**Keywords:** 4′,5,7-trihydroxyflavone, apigenin, brain, cancer, flavonoids, natural products, neurodegenerative diseases, neuroinflammation, nutraceutical, phytochemical

## Abstract

Neuroinflammation, a hallmark of various central nervous system disorders, is often associated with oxidative stress and neuronal or oligodendrocyte cell death. It is therefore very interesting to target neuroinflammation pharmacologically. One therapeutic option is the use of nutraceuticals, particularly apigenin. Apigenin is present in plants: vegetables (parsley, celery, onions), fruits (oranges), herbs (chamomile, thyme, oregano, basil), and some beverages (tea, beer, and wine). This review explores the potential of apigenin as an anti-inflammatory agent across diverse neurological conditions (multiple sclerosis, Parkinson’s disease, Alzheimer’s disease), cancer, cardiovascular diseases, cognitive and memory disorders, and toxicity related to trace metals and other chemicals. Drawing upon major studies, we summarize apigenin’s multifaceted effects and underlying mechanisms in neuroinflammation. Our review underscores apigenin’s therapeutic promise and calls for further investigation into its clinical applications.

## 1. Introduction

In many pathologies, such as neurodegenerative diseases or cerebrovascular diseases, neuroinflammation is one of the activated pathways, very often associated with cell death or oxidative stress. Neuroinflammation occurs when cells begin to produce signaling molecules (such as cytokines) to alert the body to danger (infection, toxic metabolites, autoimmune disease, etc.) in the nervous system, triggering a cascade of signals to limit damage. Targeting neuroinflammation would be strategically important in managing the various pathologies concerned. In some cases, such as Alzheimer’s disease, the question arises as to whether neuroinflammation is a cause or a consequence of neurodegeneration. Alongside this, the search for effective drug therapies exploring molecules of natural origin to enhance drug effects or mitigate side effects would be intriguing. That is why research into nutraceuticals is growing. Nutraceuticals are substances derived from food or plants that offer medical benefits. Patients are seeking a natural, effective, and safe way to treat their pathologies. Conventional therapies do not always provide the answer, and some are costly, not to mention the side effects that are sometimes associated with them. Human nutrition can be considered a solution to the problem, mainly through the inclusion of functional foods or nutraceuticals that can be used as antioxidant/anti-inflammatory agents to prevent and mitigate acute and chronic oxidative damage and inflammation.

Numerous research studies have highlighted the benefits of using dietary derivatives such as resveratrol in several human disease models, including cardiac and neurological diseases, nephroprotection, immune regulation, diabetes, obesity, and age-related diseases. Among nutraceutical options, we have chosen to target apigenin. Apigenin is widely available in plants: vegetables (parsley, celery, onions), fruits (oranges), herbs (chamomile, thyme, oregano, basil), and some beverages (tea, beer, and wine). Numerous studies have reported that apigenin exhibits various pharmacological functions and has the potential to be a therapeutic agent for inflammation and neurodegenerative-related diseases, as well as autoimmune function, and even several types of cancers [1]. Apigenin has a wide range of biological effects, all equally important for the prevention and treatment of inflammation. In this review, we focus on apigenin and neuroinflammation. After introducing them, we will explore their relationship.

## 2. Apigenin

### 2.1. Chemistry

Polyphenols are classified into different families: phenolic acids, which include hydrobenzoic acids (protocatechuic acid, gallic acid) and hydroxycinnamic acids (coumaric acid, caffeic acid, ferulic acid, curcumin); non-flavonoids, which include stilbenes (resveratrol) and lignans (secoisolariciresinol); and finally, flavonoids [2]. Flavonoids include flavonols (kaempferol, quercetin, myricetin), isoflavones (daidzein, genistein), flavanones (naringenin, eriodictyol, hesperetin), anthocyanidins (pelargonidin, cyanidin, delphinidin, petunidin, malvidin), flavonols (catechins, gallocetechin), and flavones (luteolin). Apigenin belongs to this last class of polyphenols.

**Figure 1:**
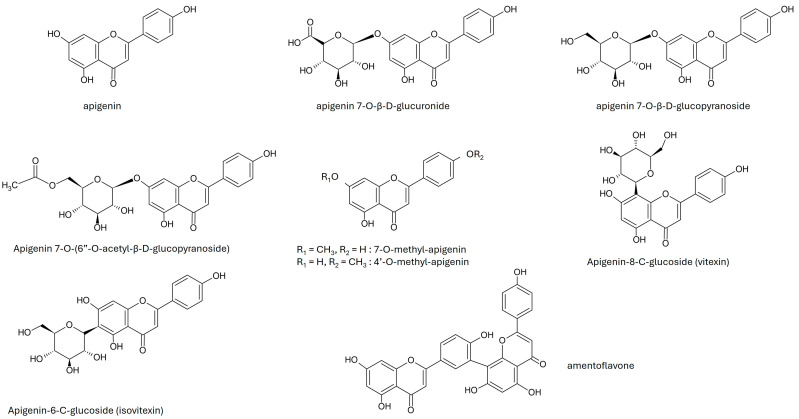
Structure of apigenin and derivatives.

Apigenin (API) is a 4′,5,7-trihydroxyflavone, based on the skeleton of 2-phenylchromen-4-one (2-phenyl-1-benzopyran-4-one) (Figure 1). In plants, apigenin is present in the form of aglycone and/or its C- and O-glycosides (detected as 6-C and 8-C-glucoside, and 7-O-glucoside), glucuronides, O-methylethers and acetyl derivatives (Figure 1) [1]. One hypothesis concerning the origin of apigenin is that free apigenin is a product of the post-harvest degradation process [3,4]. In plants, API can exist in a wide range of different glycoside forms, the presence and ratio of which are influenced by genetic background, environmental growth conditions, developmental stages, etc. [5,6]. In addition to glycosylation, in some plants, APIs form dimeric molecules to form bioflavonoids and other diverse structural groups.

**Figure 2:**
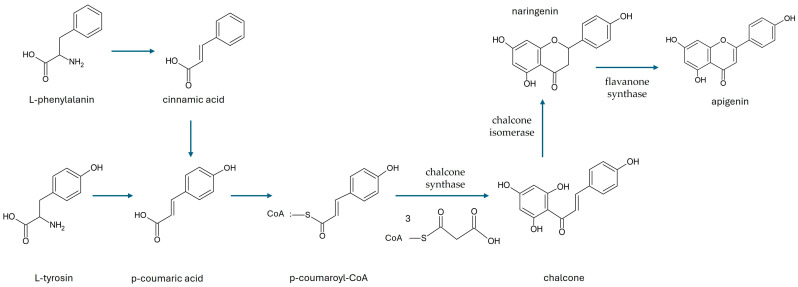
Biosynthesis of apigenin from phenylalanine and tyrosine.

Apigenin is biosynthesized in the cytoplasmic surface of the endoplasmic reticulum, and the reaction is catalyzed by a series of enzymes. Biogenetically, apigenin is a product of the phenylpropanoid pathway and can be obtained from phenylalanine and tyrosine, two precursors derived from shikimate (Figure 2). From phenylalanine, cinnamic acid is formed by non-oxidative deamination followed by C-4 oxidation, which is then converted to p-coumaric acid. From tyrosine, p-coumaric acid is formed directly by deamination. After activation with CoA, p-coumarate is condensed with three malonyl-CoA residues and then aromatized by chalcone synthase to form chalcone, which is then isomerized by chalcone isomerase to form naringenin. Finally, a flavanone synthase oxidizes naringenin to apigenin [1].

API is rarely found in its free form. Its formation is generally followed by the action of certain glycosyltransferases, hydroxyltransferases, and methyltransferases which catalyze the methylation and hydroxylation of API to form various derivatives. An efficient method for the in vitro synthesis of apigenin glucosides is the glycosylation reaction using the uridine diphosphate-glucosyltransferase YjiC, from Bacillus licheniformis DSM 13 [7]. Other methods exist for synthesizing apigenin, such as microwave irradiation of the ketoester used as a starting material [8,9] or phloroglucinol [10,11]. A wide range of different synthetic API derivatives are also synthesized as potential pharmacologically active compounds [12,13].

### 2.2. Sources

**Figure 3:**
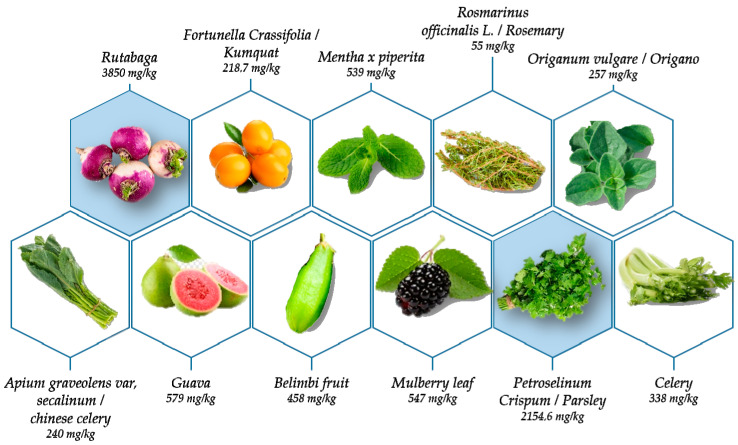
Source of apigenin. Numerous fruits, vegetables, and aromatic plants contain apigenin, particularly rutabaga and fresh parsley, at levels of 3850 mg/kg and 2154.6 mg/kg, respectively.

Apigenin, in free or conjugated form, is widespread in the plant kingdom in both edible and medicinal plants, making it an important component of the Mediterranean diet [14,15]. Apigenin can exist in a wide range of different glycoside forms whose presence and ratio are influenced by genetic background, environmental growth conditions, developmental stages, etc. [16]. This compound can be found in significant quantities in vegetables, fruit, herbs, cereals, and herbal drinks. The United States Department of Agriculture (USDA) database has inventoried the flavonoid content of 506 food products that include apigenin [17]. In this database, the quantities mentioned are average values expressed in mg/100 g of edible portion. The highest amount of API identified in fresh parsley was 215.46 mg/100 g Formula Weight (FW) (Figure 2). Other foods containing API are celery (i.e., Chinese celery, with 24.02 mg/100 g), kumquat (21.87 mg/100 g), and rutabaga (3.85 mg/g edible portion) (Figure 2). Apigenin is also found in spices such as oregano, mint, rosemary, sage, and thyme. Sixty-two edible plant species were studied by Miean and Mohamed [18]. After the extraction and hydrolysis of flavonoid glycosides, 11 of them contained apigenin. The highest amount was found in guava (579.0 ± 0.02 mg/kg), followed by mulberry leaf (547.0 ± 0.07 mg/kg), belimbi fruit (458.0 ± 0.04 mg/kg), and celery (338.5 ± 0.04 mg/kg) (Figure 3) [18]. Apigenin has been identified as an active ingredient in *Scutellaria barbata* D. Don (Lamiaceae) [19], *Castanea sativa* Mill. (Fagaceae) [20], *Portulaca oleracea* L. [21], *Marrubium globosum* ssp. Libanoticum [22], *Combretum erythrophyllum* (Combretaceae) [23], *Aquilegia oxysepala* [24], and propolis [25], most of which are traditional medicinal plants or alternative medicines. Other natural sources of apigenin exist. For example, *B. pendula*, *Rosa laevigata* Michx., and *M. chamomilla* also contain it.

Glycosylation is not the only process that exists; in some plants, apigenin dimerizes to form bioflavonoids and other diverse structural groups. The best-studied apigenin dimer of greatest pharmacological interest is amentoflavone (3′, 8″-biapigenin) found in well-known medicinal plants such as St. John’s wort (*Hypericum perforatum* L.), gingko (*Ginkgo biloba* L.), and selaginella (*Selaginella* sp.) [26].

## 3. Neuroinflammation

Neuroinflammation, inflammation of the brain and neurons that comprise the nervous system, is a cerebral immune response mechanism designed to protect the brain from aggression. It is an essential, innate function that the organism has developed to protect itself. Under normal circumstances, microglia detect changes in the environment and moderate disruption in homeostasis. In the event of injury, infection, toxin exposure, aging, autoimmune disease, or neurodegenerative disease, microglia increase inflammatory signals, such as cytokines, chemokines, leukotrienes, and prostaglandins. Reactive oxygen species and secondary messengers produced by the resident glial cells, including microglia, astrocytes, endothelial cells, and peripheral immune cells, are also elevated. These neuroinflammatory responses have immune, physiological, biochemical, and psychological consequences.

Neuroinflammation is not a universal mechanism, and depends on the context, duration, and evolution of the initial stimulus. Indeed, inflammation can lead to the recruitment of immune cells, edema, tissue damage, and, probably, cell death. Neuroinflammation plays a role in the progression of many neurodegenerative diseases, including multiple sclerosis and amyotrophic lateral sclerosis, and in the development of chronic pain, but its role is complex. The brain is a special tissue, with a very limited capacity for renewal, which makes it essential to limit the damage inherent in any inflammatory and immune reaction. Immune reactions in the brain are just as robust and effective as those in the periphery, but they have their own actors and characteristics.

We will start by focusing on the blood–brain barrier that surrounds and protects the brain, then move on to microglia, the main players in the immune response and inflammation, with their various links to signaling pathways (receptors involved) and soluble factors (cytokines, chemokines).

### 3.1. Blood–Brain Barrier

**Figure 4:**
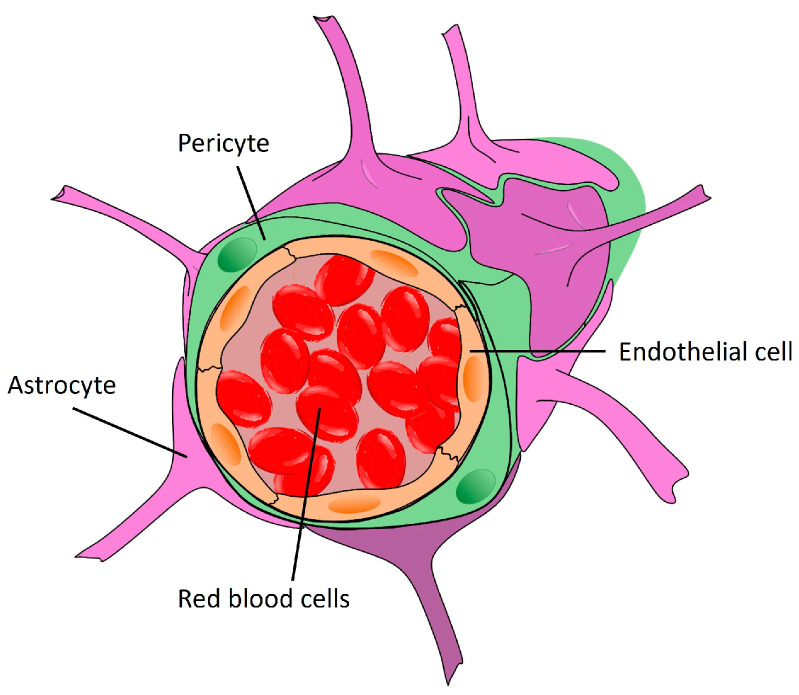
The structure of the blood–brain barrier. The BBB consists of a continuous layer of contiguous endothelial cells resting on a basal lamina encrusted with pericytes and firmly sheathed by astrocyte roots, which almost completely cover the surface of cerebral microvessels.

The first and most obvious structure involved in neuroinflammation is the blood–brain barrier (BBB), which anatomically separates the brain parenchyma from circulation [27]. It maintains cerebral homeostasis by limiting molecular diffusion and cell migration, while ensuring nutrient supply via specific transport systems. Endothelial cells form tight junctions between themselves, and are affixed to a very thick basement membrane, which limits paracellular flow. They also have a low capacity for endocytosis, which limits transcellular flow. Surrounding the vessels are pericytes and perivascular macrophages, as well as astrocytic feet ensuring the integrity of the structure (Figure 4). This concerns most of the brain. In certain areas, the vessels are looser, making the barrier more permeable. For example, in the arcuate nucleus, the BBB is permeable due to the presence of fenestrated blood vessels [28,29,30]. In certain pathologies, the BBB undergoes changes and can become more permeable. It is important to underline that the physiological properties of the BBB can be changed under the action of molecules such as oxysterols resulting from cholesterol oxidation, which can be increased in several diseases including neurodegenerative diseases [31,32].

### 3.2. Microglia

**Figure 5:**
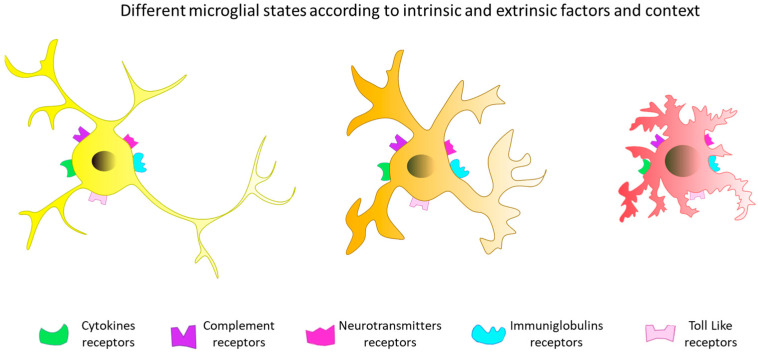
Some microglial states. Microglial cells are highly dynamic cells that constantly survey the brain environment. Thanks to their multiple surface receptors, they can adapt their morphology, ultrastructure, and molecular profile in response to a wide range of stimuli.

When we talk about the main cellular players, microglia are the first to be mentioned, as these cells are the major immunocompetent cells of the CNS. Microglia can have different origins depending on the condition. In physiological conditions, during development, microglial cells invade the CNS of the embryo. The progenitor cells of microglial cells are derived from primitive mesodermal hematopoiesis. In pathological conditions, microglial cells may also be of monocytic origin. Pio Del Rio-Hortega was the first to identify microglial cells in the CNS. He described a small population of phagocytic cells with the ability to migrate and defined them as “migratory cells”. He then postulated that they were of mesodermal origin (for review: [33]). He distinguished them from the neurons, astrocytes, and oligodendrocytes making up the “macroglia” [34]. Later, in 1999, Alliot et al. observed microglial cells at embryonic stage E9.5 in the rodent brain and suggested that the cell precursors originated in the yolk sac, formed from the mesoderm and endoderm [35]. The yolk sac is the site of hematopoiesis, where primitive macrophages differentiate. These macrophages are released into the bloodstream and colonize tissues. The neuroepithelium is then the first organ colonized at E9.5; embryonic microglia are formed and will then expand, colonizing the whole brain and maintaining themselves throughout life [36]. After birth, the microglial population continues to expand, suggesting a different origin for embryonic microglial cells. Numerous studies have proposed that circulating bone marrow-derived monocytes could be the origin of microglial cells colonizing the brain in the postnatal period [37,38]. However, studies by Ginhoux and Kierdorf’s team demonstrate that microglial cells originate exclusively from an embryonic source without recourse to circulating monocytes [39,40]. Indeed, at E13.5 in rodents, the BBB begins to build up and isolates the brain from monocyte colonization [36]. Only in special circumstances, when embryonic microglia are eradicated or when the BBB is damaged, have circulating monocytes been shown to infiltrate the CNS [33,41]. Microglial cells account for around 10–15% of glial cells and are distributed throughout all brain regions [42]. Microglial density is highest in the cortex, followed by the limbic system, basal ganglia, diencephalon, brainstem, and cerebellum [43]. In physiological and pathological conditions, microglial cells are not described in the same way (Figure 5). Microglial cells were initially considered to be dormant immune cells that activate only in response to pathological events in the adult brain (for review: [44]). However, it is now accepted that adult microglial cells are highly dynamic cells playing important roles in the maintenance of brain homeostasis. For many years, microglia have been described as existing in an M1 or M2 state, transitioning from a resting state to an activated state. However, evolving scientific data suggest that microglia are consistently active. They respond in a constant but situation-dependent manner to changes in their CNS environment, even in physiologically normal conditions. Depending on life stages, CNS region, species, gender, and health/disease context, microglia will react by adopting different behaviors and performing different functions [45]. Microglia can produce numerous pro-inflammatory cytokines such as interleukin (IL)-1β, tumor necrosis factor (TNF)-α and IL-6, inducible nitric oxide synthase (iNOS), and cyclooxygenase (COX)-2 [46,47] and express the surface marker CD86 [48], but also anti-inflammatory ones such as IL-10, IL-1ra, and transforming growth factor (TGF)-β [49]. For example, in a state of neuroprotection and tissue repair, microglia synthesize anti-inflammatory cytokines such as IL-4, IL-10, IL-13, or TGF-β [49]. This phenotype also participates in the elimination of cellular and molecular debris by phagocytosis [46]. Microglial cells are characterized by the expression of the surface markers CD206 and CD36 [47,49].

**Figure 6:**
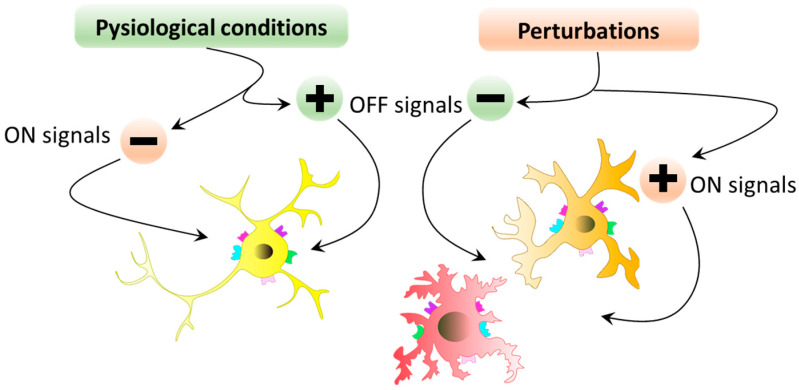
“ON”/”OFF” signals. Under physiological conditions, OFF signals predominate, and microglial cells exist in a basal, homeostatic state. If there are perturbations, ON signals replace the dominant OFF signals, and microglial cells adapt their state accordingly.

The immune functions of microglial cells are controlled by two classes of signals known as “on” and “off” signals [50] (Figure 6).

**Figure 7:**
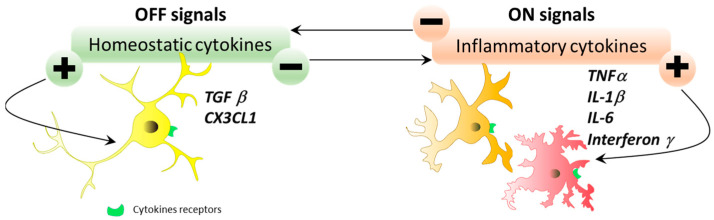
“Homeostatic cytokine”/“inflammatory cytokine” signals. Microglial cells exist in a basal state when transforming growth factor beta (TGF-β) and CX3CL1 are the dominant molecules in their environment. However, if inflammatory cytokines become predominant (TNFα, IL1β, IL-6, or interferon γ), microglial cells can detect these changes and adapt their state accordingly.

“Off” signals are constitutively expressed in the brain under physiological conditions. The disappearance of these signals can lead to the creation of a warning signal inducing microglial state change. The maintenance of microglial cells in a basal state is ensured by “off” signals. Conversely, “on” signals are produced when abnormal conditions arise, to initiate microglial cell state change. These environmental variations are detected by the variety of receptors expressed by microglial cells. Various communication pathways are involved in this “on”/”off” signaling and participate in the dialog between microglial cells and their environment. Microglial cells express a first class of receptors: receptors for neurotransmitters such as glutamate, gamma-aminobutyric acid (GABA), noradrenaline, purines, and dopamine. These receptors detect neurotransmitter concentrations in the microglial environment and adapt the microglial response to changes in concentration. Indeed, changes in neurotransmitter levels are present in certain pathologies, leading to excitotoxicity and microglial response (state change). Another major family of microglial cell membrane receptors are cytokines and chemokines. Under physiological conditions, cytokines participate in sleep regulation, various endocrine functions, neuronal development, and normal aging [51]. In pathological conditions, cytokines inform immune cells of abnormalities appearing in the parenchyma to enable the development of an appropriate immune response. In inflammatory conditions, cytokine production and cytokine receptor expression are increased. It is important to note the existence of inflammatory cytokines whose expression is induced during inflammatory conditions (“on” signal) and homeostatic cytokines whose expression is constitutive (“off” signal). On the other hand, the cessation of homeostatic cytokine expression constitutes an “on” signal of microglial state change. Microglial cell state depends on the fine homeostatic regulation of cytokine production (balance of “on” and “off” signals). Examples of “off” signals are TGFβ or CX3CL1/fractalkine, while “on” signals are TNFα, IL1β, IL-6, or interferon gamma (Figure 7). The third family of Toll-like receptors belongs to the pattern-recognition receptor (PRR) family, which recognizes pathogen-associated molecular patterns (PAMPs) and can initiate immune responses [52]. 

**Figure 8:**
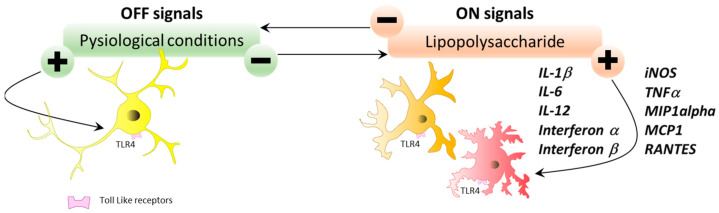
“OFF”/“LPS” signals. In response to LPS, microglial cells, through the TLR4 receptor, modulate their state and produce a variety of molecules, such as IFNα, IFNβ, IL-1β, IL-6, IL-12, TNFα, iNOS, MIP-1α, MCP-1, and RANTES.

During cellular damage or stress, Toll-like receptors (TLRs) also detect endogenous ligands released under these conditions, known as DAMPs (danger-associated molecular patterns) and influence the inflammatory response. These TLRs bridge the innate response (through pathogen detection and induction of the inflammatory response) and the adaptive immune response (through pathogen detection and induction of the inflammatory response) with the adaptive immune responses (by recruiting and activating cells of the adaptive system). Microglial cells express all known members of the TLR family identified to date. Each TLR has its preferred ligand, and activation of a TLR triggers a particular inflammatory response [53,54]. For example, LPS activates TLR4 and triggers a response involving IFNα, IFNβ, IL1β, IL-6, IL-12, TNFα, iNOS, Macrophage inflammatory protein 1α (MIP-1α), monocyte chemoattractant protein 1 (MCP1), and RANTES (Regulated on Activation, Normal T cell Expressed and Secreted) (Figure 8).

The fourth family consists of immunoglobulin receptors such as CD200, Signal Regulatory Protein α (SIRPα), and Triggering Receptor Expressed on Myeloid cells 2 (TREM2). In the immune system, complementation is the first line of defense against infection, rapidly eliminating incoming pathogens and regulating the immune response. In addition, the complementation system eliminates modified self-cells such as apoptotic cells and cellular debris to protect against autoimmunity. Circulating complementary proteins are inactive until they associate with a cell membrane. Complementation facilitates phagocytosis and the removal of labeled cellular material and is also thought to play a role in regulating cytokine production and chemotaxis. In neurodegenerative diseases, there is high expression of complementary components, as well as increased neuronal death induced by numerous molecular and cellular defects. Neuronal death is believed to be preceded by aberrant synaptic function and extensive synaptic loss [55]. These dysfunctions and synaptic losses could lead to neuronal death and disease progression. Complementation would mark these dysfunctional synapses for elimination, and activated microglia would phagocytose these elements [55]. This process, in which microglia selectively eliminate synapses from damaged neurons, is known as “synapse stripping” [56]. In Alzheimer’s disease, for example, synapses are severely dysfunctional and lost from the onset of the disease. Moreover, this disease is characterized by strong microglial state change and increased expression and activation of complementary components [57,58]. The sixth family is that of major histocompatibility complex class I (MHC I) receptors, which are thought to play a greater role in synapse elimination during development [59]. Thanks to their structural and functional characteristics, microglia can be active both physiologically and under pathological conditions. In physiological conditions, microglia provide dynamic and constant surveillance of the brain parenchyma. Microglial cells have highly branched, mobile extensions that are constantly in a state of retraction or extension. This enables microglia to rapidly detect abnormal changes in their environment, such as infection, injury, neurodegeneration, and changes in intense neuronal activity. This function would involve some of the receptors mentioned above: purinergic signaling mediated by microglial P2Y12 receptors [60,61] and the neuron/microglia signaling pathway, fractalkine/CX3CR1 [62]. Microglia would have periods of high mobility, as mentioned above, but also pauses (4–5 min) during which they would eliminate accumulated metabolic products and damaged tissue compounds. During this static phase, microglia interact with other cell types (astrocytes, neurons, and blood vessels). Microglial cells also modulate synaptic plasticity in the mature CNS, as well as synaptic activity [63] and adult neurogenesis [64,65]. As mentioned above in the case of the physiological functions performed by microglia, motility and rapid movement enable microglia to travel to an injured site. It is generally the microglial cells close to the lesion that become activated, while those further away (>90 µm) either fail to respond or become so later. Either the entire microglial cell is displaced, or only individual extensions [66,67,68]. Signaling pathways have been described involving ATP (release from damaged cells, astrocytes) and microglial P2Y12 receptors [60]; the nitric oxide (NO)/cyclic guanosine monophosphate (cGMP) pathway for microglial cytoskeleton utilization [67], or cell signaling involving chemokines (CXCL10/CXCR3) [69].

### 3.3. Example of Neuroinflammation Process in Alzheimer’s Disease

To better understand the mechanisms involved in pathologies, we can examine Alzheimer’s disease. In the pathological context of Alzheimer’s, peripheral and central immune cells, particularly microglia, are key players. Indeed, microglial activation seems to participate in the initiation of Alzheimer’s disease [70].

Microglial cells produce TGF-β, an inflammatory cytokine involved in numerous processes, including immune response and inflammation. In the context of the CNS, it can be either pro-inflammatory or neuroprotective. In Alzheimer’s disease, TGF-β participates in the activation and recruitment of microglia and astrocytes to Aβ plaques, potentially aiding in plaque removal. However, dysregulation of TGF-β signaling may lead to neuroinflammation and neurodegeneration [71]. Some studies highlight microglial and astrocytic activation in cerebral inflammatory disorders on post-mortem tissue. In this state, microglial cells secrete chemokines, cytokines, and inflammatory factors such as TNF-α, IL-6, and IL-1β, which increase inflammation and neurodegeneration [72].

Microglial and astrocytic reactions depend on microglial interactions with tau and amyloids, neuronal damages and pro-inflammatory messengers, but tau and neurodegeneration can induce microglial disruption independently of amyloids [73].

Neuroinflammation can initiate these pathological changes. Astrocytes and microglial activation could be the drivers of self-amplification [74].

Furthermore, the blood–brain barrier is often disrupted in Alzheimer’s disease and can lead to increased inflammation through peripheral T cells’ infiltration, and to inflammation and neuronal damage [75].

However, it is important to remember that there is a huge heterogeneity of neuroinflammatory profiles in Alzheimer’s disease. Many factors influence neuroinflammation in this pathology, including infectious and microbial burden, complex genetic patterns, the integrity of the gut microbiota, aging, a history of acute or chronic neuroinflammatory conditions (such as vascular lesions or psychiatric disorders), or a history of diseases associated with systemic inflammation (such as obesity or diabetes). Nonetheless, further research is needed to fully understand the relationships between different combinations of these factors and the development of Alzheimer’s disease.

## 4. Impact of Apigenin on Neuroinflammation in Pathologic Cases

**Figure 9:**
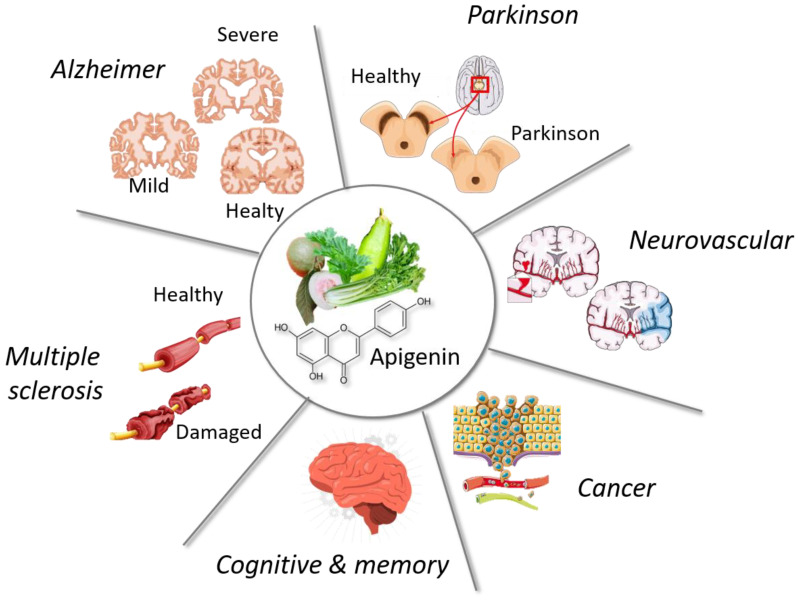
Apigenin and links with neuroinflammation in a variety of pathologies.

Neuroinflammation is present not only in neurodegenerative diseases, but also in brain cancers, cardiovascular disease, cognitive/memory disorders, and metal/chemical poisoning. Apigenin has been tested on these different pathologies (Figure 9), and Mushtaq et al. describe experimental evidence and mechanisms for therapeutical properties of apigenin in their recent review [76].

As previously mentioned, our work focuses on neuroinflammation, so searches were carried out using the keywords “apigenin” and “neuroinflammation” for original articles in the Pubmed database. No clinical trials dedicated to neuroinflammation and apigenin were found after searches on Pubmed/Clinical trials and clinicaltrials.gov. On clinicaltrials.gov, four studies concern apigenin and its absorption/metabolism/excretion (no results posted, but one article), apigenin and septic shock (no results posted), apigenin and Parkinson’s disease (under recruitment, no associated publications) and one withdrawn. On Pubmed, 23 articles concern clinical trials, including 1 in common with clinicaltrials.gov about apigenin absorption/metabolism/excretion, but none concern neuroinflammation. These articles deal with aging, migraine, anxiety, cancer (ovarian, colorectal, breast), the presence of apigenin in urine, and mechanisms such as apoptosis and oxidative stress.

### 4.1. Neurodegenerative Diseases

Neurodegenerative diseases (Alzheimer’s disease (AD), Lewy body disease, Parkinson’s disease, Huntington’s disease, posterior cortical atrophy, amyotrophic lateral sclerosis (ALS), or multiple sclerosis (MS)) are chronic progressive diseases that affect the nervous system. Their frequency increases significantly with age. It is estimated that there are currently over a million people in France affected by Alzheimer’s disease (850,000 people) and other dementias; around 200,000 people treated for Parkinson’s disease and 100,000 people for multiple sclerosis; and around 2300 new cases a year of motor neuron disease, the main cause of which is amyotrophic lateral sclerosis (sante.gouv.fr). Neurodegenerative diseases are a major cause of disability, dependency, institutionalization, and hospitalization. They have a major impact on the quality of life of sufferers, their families, and their careers. At present, the treatments available are purely symptomatic and of variable effectiveness. It is in this context that several studies have been carried out using derivatives from food, such as apigenin.

#### 4.1.1. Multiple Sclerosis

Multiple sclerosis is an autoimmune disease: the immune system attacks the myelin sheath that surrounds axons in the central nervous system. This leads to scattered lesions in the central nervous system, known as plaques [77]. These lesions are the site of inflammation, demyelination, and, often, axonal degeneration. Several experimental models are used to study MS. These can generally be classified into three broad categories: experimental autoimmune encephalomyelitis (EAE) models, virus-induced inflammatory demyelination models, and toxic demyelination models [78]. In an EAE model, infiltration of dendritic cells (DCs) and T cells into the CNS results in the production of pro-inflammatory cytokines TNF-α, IL-6, IL-17, IL-1β, and IFNγ, leading to neurodegenerative effects associated with neuroinflammatory diseases. Apigenin has been shown to inhibit cell surface expression of co-stimulatory molecules as well as certain dendritic cell functions such as pro-inflammatory cytokine production and T-cell differentiation [79,80]. Apigenin can also block LPS-induced lethality in vivo and pro-inflammatory cytokine expression via NF-κB inactivation (suppression of p65 phosphorylation) [81]. Apigenin also inhibits COX-2 enzyme activity and monocyte adhesion to the human umbilical vein endothelium by reducing the presence of cell adhesion molecules such as Vascular cell adhesion protein 1 (VCAM-1), Intercellular adhesion molecule 1 (ICAM-1), and E-selectin [82,83]. Apigenin would thus have the capacity to inhibit the entry of immune cells into the CNS and prevent neuroinflammation, given that the molecules play an essential role in controlling leukocyte migration through endothelial cells, including those of the blood–brain barrier. Some authors have also demonstrated apigenin’s ability to reduce the expression of iNOS, nitric oxide, and prostaglandin E2 (PGE2) production in microglial cells and macrophages, showing potential neuroprotective effects [84]. In mice with active or relapsing EAE, apigenin significantly reduces disease severity and recurrence by reducing immune infiltration in the central nervous system. A shift from a pro-inflammatory to a more tolerogenic phenotype is also observed, due to the significant reduction in the cell surface expression of MHC class II molecules and CD86 on dendritic cells [85]. Apigenin also restores immune balance by significantly reducing the number of Th17 cells and increasing the phenotype of regulatory T cells. Apigenin may therefore play a part in reducing neuroinflammation and the clinical pathologies associated with it in MS [85]. In another study, the same team showed that apigenin exerts its effects by shifting CD-modulated T-cell responses from Th1 and Th17 to Treg-directed responses, given the decreased expression of T-bet, IFN-γ (Th1), and IL-17 (Th17) and the increased expression of IL-10, TGF-β, and forkhead box P3 (FoxP3) (Treg) in cells from normal human donors and EAE-affected mice [86]. RelB, a protein of the NF-κβ pathway, plays a central role in DC maturation and antigen-presenting capabilities and DC-mediated T-cell activation. Apigenin reduces RelB mRNA and protein levels as well as its nuclear translocation. Furthermore, the effects of apigenin on RelB were potentiated following siRNA-mediated inhibition of RelB, confirming its role in apigenin-mediated regulation of DC biology [86].

The information in this paragraph is summarized in Table 1.

#### 4.1.2. Parkinson’s Disease

Apigenin has also been tested in Parkinson’s disease, a neurodegenerative disease characterized by a loss of dopaminergic neurons in the substantia nigra pars compacta associated with oxidative stress, neuroinflammation, and cell death. In a rotenone-induced parkinsonian rat model where there is a loss of tyrosine hydroxylase (TH) immunoreactivity in the striatum and substantia nigra, apigenin treatment reduces α-synuclein aggregation and increases TH protein expression as well as dopamine D2 receptor (D2R) expression [89]. Significant attenuation of increased NF-κB gene expression in the rotenone-induced group and inhibition of neuroinflammation in the substantia nigra pars compacta are also observed with inhibition of rotenone-induced release of the pro-inflammatory cytokines TNF-α and IL-6 and the pro-inflammatory enzyme iNOS-1 [89]. In the experimental LPS model in rats (unilateral intranigral injection), oral administration of apigenin alone or in combination with piperine and with bromocriptine was performed to assess its effects on LPS injection. LPS has been shown to induce microglial state change, neuroinflammation, oxidative stress, and altered neurotransmitters, with symptoms similar to those of Parkinson’s disease [90]. A significant change in body weight and behavioral parameters assessed on a weekly basis was observed after LPS injection, as well as increased levels of nitrite, malondialdehyde (MDA), SOD, TNF-α, IL-1β, IL-6, and caspase-1, and decreased levels of CAT, glutathione content (GSH), and complex-I. LPS treatment also induced altered neurotransmitter levels (dopamine (DA), GABA, and glutamate) and cellular changes [90]. Significant attenuations of altered body weight, motor deficits, oxidative stress, neuroinflammation, and neurotransmitters in rat brains are observed following treatment with apigenin alone or in combination with piperine [90]. This neuroprotective activity is thought to involve modulation of the NF-kB and Nrf2 signaling pathways in the striatum [90]. In a 1-methyl-4-phenyl-1,2,3,6-tetrahydropyridine (MPTP)-induced model of aged male C57BL/6 mice, apigenin administration was shown to attenuate MPTP-induced histopathological changes in brain tissue and reversed changes in the expression and concentrations of TNF-α, IL-1β, IL-6, IL-10, and TGF-β [91].

The information in this paragraph is summarized in Table 2.

#### 4.1.3. Alzheimer’s Disease

Alzheimer’s disease is caused by a slow degeneration of neurons. Neuronal degeneration first affects neurons in the hippocampus, leading to impairment of short-term memory, and then gradually spreads throughout the brain. Two proteins are involved: (1) amyloid beta peptide, which accumulates abnormally, forming plaques known as amyloid plaques or “senile” plaques (this accumulation is toxic to nerve cells in affected patients) and (2) tau protein, which is altered in affected patients, causing successive disorganization of neurons, accumulation of filaments inside them (neurofibrillary degeneration), and then nerve cell death [92]. Degeneration occurs very slowly, and it can take years for symptoms to appear. The effect of apigenin has been tested at the molecular level, in signaling pathways and in cognitive functions. In an Alzheimer’s disease model, SH-SY5Y neuronal cells, apigenin decreased Caspase-3/7 activity and thus prevented cell death by apoptosis [93].

Co-cultures of neurons and glial cells, obtained from the cortex of newborn and embryonic Wistar rats, were exposed to lipopolysaccharide, or IL-1β or Aβ oligomers, then treated with apigenin [94]. In this situation, apigenin has several effects: preservation of neuron and astrocyte integrity, reduction in microglial response, induction of increased brain-derived neurotrophic factor (BDNF) expression, and modulation of inflammatory cytokine mRNA expression as well as a reduction in OX42, IL-6, and gp130 expression. These elements support the use of apigenin as an important neuroimmunomodulator agent for the treatment of neurodegenerative diseases via neuroprotective and anti-inflammatory effects [94].

Apigenin may also have effects on cognition. In a passive avoidance task in male Wistar rats, the use of a high dose of apigenin improves long-term memory [95]. Chronically, apigenin decreases Morris Water Maze cognitive impairment in rodent models of diabetes [96] and isoflurane-induced cognitive dysfunction [97]. The neuroprotective effects of chronic apigenin use were seen in the amyloid precursor protein/presenilin 1 (APPxPS1) AD mouse model [98]. Chronic treatment with apigenin over 3 months improved learning and memory in the Morris Water Maze in APPxPS1 mice, and reduced fibrillar amyloid deposits as well as insoluble β-amyloid concentrations [98]. In a GFAP-IL6 (glial fibrillary acidic protein-interleukin 6) transgenic mouse model, a model exhibiting neuronal loss and atrophy, chronic activation of microglia and astrocytes, increased expression of inflammatory mediators [99], and BBB disruption and age-related motor and cognitive impairment [99,100,101], apigenin decreased the number of Iba-1+ microglia in the hippocampus of GFAP-IL6 mice and altered microglial morphology, but no reversal of spatial memory impairment was observed [102]. In computational and experimental studies by Shahwan et al. targeting Alzheimer’s disease, apigenin was shown to occupy the iron-binding pocket of human transferrin [103]. The authors were interested in the transferrin/apigenin complex because iron dyshomeostasis plays an important role in maintaining the neuroinflammatory phenotype, demonstrating the importance of maintaining iron balance [103]. The binding between apigenin and transferrin is relatively stable, so this could open new therapeutic opportunities using apigenin to control iron homeostasis and, hence, neuroinflammation.

*B. pendula* leaf extract, composed of high quantities of polyphenolic carboxylic acids (gallic, chlorogenic, caffeic, trans-p-coumaric, ferulic, and salicylic acids) and flavonoids (apigenin, luteolin, luteolin-7-O-glucoside, naringenin, hyperoside, quercetin, and quercitrin) was orally administered to Wistar rats dosed with Aβ1-42 [104]. This B. pendula leaf extract decreased lipid peroxidation and neuroinflammation (IL-1β decreased), modulated the expression of specific proteins (NF-κB active form increased, COX2 negatively regulated), increased antioxidant capacity (Superoxide Dismutase (SOD), Catalase (CAT) and GPX activity increased), and improved spontaneous alternation behavior.

Aluminum, a potent environmental neurotoxin linked to neurodegenerative disorders, is present in water, leading to bioaccumulation in aquatic organisms and ultimately in humans. In contrast, increased physical activity is known to have a positive effect on neurodegenerative diseases. Researchers have therefore studied the combined effect of physical exercise and intake of the nutraceutical apigenin on a zebrafish model treated with aluminum. These fish developed anxiety, increased aggression, unusual swimming habits, and memory impairment, characteristics found in Alzheimer’s disease [105]. The combination of exercise and apigenin improved anxiety, memory loss, and aggression, and increased levels of antioxidant enzymes and acetylcholinesterase (AChE) activity. In addition to these improvements, the physical activity/apigenin combination counteracts increased expression of genes linked to neuroinflammation and Alzheimer’s disease [105].

The information in this paragraph is summarized in Table 3.

### 4.2. Cancer

When a tumor forms, a process of angiogenesis takes place to enable the tumor to develop and metastasize. Endothelial cells are recruited, proliferate, and differentiate to form endothelial tubes and capillaries. These vascular structures supply nutrients and oxygen to the tumor and eliminate its metabolic products. In addition to tumor angiogenesis, there is a reduction in tumor apoptosis, an increase in invasion and metastasis, immune suppression, and inflammation associated with tumor development. In parallel, an increase in pro-inflammatory markers such as COX-2 (which converts arachidonic acid in prostaglandin H2) is also detected [106]. To promote tumor invasion, the blood–brain barrier is broken by the action of metalloproteinases (MMPs), particularly MMP-9, secreted by brain tumors. In this context, the effectiveness of flavonoids on inflammation and the formation of new capillaries has been tested. Fisetin, apigenin, and luteolin have been shown to inhibit in vitro capillary structure formation by human brain microvascular endothelial cells (HBMECs) [107]. In the same cellular model, fisetin, apigenin, and luteolin inhibited both gene expression and protein secretion of MMP-9 and the gene and protein expression of COX-2, both induced by carcinogens [107]. The signaling pathway involved in these two processes is thought to be the nuclear factor-kappa B (NF-κB) pathway. The use of apigenin could therefore reduce the disruption of the blood–brain barrier during neuroinflammation induced by the development of brain tumors.

The information in this paragraph is summarized in Table 4.

#### 4.2.1. Cardiovascular Diseases

Among cardiovascular diseases, stroke is the second leading cause of death worldwide, and one of the most frequent causes of disability in adults. During cerebral ischemia, certain mechanisms can lead to cell death, notably excitotoxicity, acidotoxicity and ionic imbalance, peri-infarct depolarization, oxidative and nitrative stress, inflammation, and apoptosis [110]. At the same time, blood reperfusion can cause severe injury. This is followed by mitochondrial dysfunction, the release of glutamate and pro-inflammatory mediators, and the production of reactive oxygen species (ROS) and/or lipid peroxidation. It has been shown that apigenin can inhibit nitric oxide production and thus protect neurons from injury in middle cerebral artery occlusion [84]. Flavonoid-rich extract (FRE) from Rosa laevigata Michx contains ten chemicals including chlorogenic acid, 4-hydroxy-3-methoxybenzoic acid, apigenin, luteolin, kaempferol, quercetin, kaempferide-3-O-glucoside, quercetin-3-rhamnoside, rutin, and isorhamnetin-3-O-β-rutinoside. This extract has all the qualities required to inhibit cell death, oxidative stress, and inflammation induced in a model of cerebral ischemia–reperfusion (I/R) injury. Indeed, regarding cell death, for example, researchers have observed a reduction in DNA fragmentation, up-regulation of anti-apoptotic proteins such as Bcl-2, and down-regulation of pro-apoptotic proteins such as p53, Apaf1, Fas, FasL, Bax, Bid, cytochrome C, and active Caspase-3, -9, and -8. In addition, FRE decreases the expression of proteins involved in the regulation of inflammation: NF-κB, iNOS, MMP-9, COX-2, TNF-α, IL-1β, IL-4, and IL-6 [108]. The ethanol extract of Verbena officinalis (VO Ex), which mainly contains hastatoside, verbenalin, acteoside, luteolin, apigenin, and hispidulin, showed, in a primary astrocyte injury model induced by oxygen–glucose deprivation/reperfusion (OGD/R), effective inhibition of the IL17A signaling pathway and attenuation of neuroinflammation [109].

#### 4.2.2. Cognitive and Memory Disorders

In cognitive impairment, neuroinflammation and abnormal histone acetylation are observed. In a model of aged rats exposed to isoflurane (impaired spatial learning and memory), deregulated acetylation of histones H3K9 and H4K12 in the hippocampus accompanied by reduced BDNF expression and suppression of the BDNF downstream signaling pathway were observed [97]. After apigenin treatment, histone acetylation and BDNF signaling were restored. Apigenin also suppressed neuroinflammation by upregulating pro-inflammatory cytokines and the NFκB signaling pathway [97].

In a scopolamine-induced rat model of amnesia, the effects of the hydroalcoholic extract of *M. chamomilla* L. were tested on memory processes. The major components identified in this extract were chlorogenic acid, apigenin-7-glucoside, rutin, cynaroside, luteolin, apigenin, and apigenin-7-glucoside derivatives. The use of this extract reversed the scopolamine-induced decrease in spontaneous alternation in the Y maze test and the scopolamine-induced increase in working and reference memory errors in the radial arm maze test [111]. In addition to memory recovery, the extract tested recovered acetylcholinesterase activity and oxidant–antioxidant balance in the rat hippocampus, as well as restoring scopolamine-diminished BDNF expression and increased IL1β expression in the same rat hippocampus [111].

It has also been shown that a higher dietary intake of certain (poly)phenols may be associated with better sleep quality in adults [112]. However, a dichotomy exists between normal-weight and overweight people. Indeed, in normal-weight people, a stronger association between certain classes, subclasses, and individual compounds and sleep quality has been demonstrated. In overweight or obese people, sleep quality was not associated with any class of dietary (poly)phenols [112].

A reduction in cognitive function is observed as the brain ages. A brain transcriptome analysis was carried out in young and aged mice receiving apigenin in their drinking water [113]. In treated aged mice, improved learning and memory was demonstrated, and transcriptomic analyses showed that genes differentially expressed with age and apigenin were related to immune responses, inflammation, and cytokine regulation [113]. Apigenin treatment in aged animals down-regulated certain genes/transcripts associated with immune activation/inflammation, and up-regulated those related to neuronal function and signaling [113]. The transcriptomic differences observed were linked in part to glial cells and astrocytes [113].

The information in this paragraph is summarized in Table 5.

#### 4.2.3. Toxicity Related to Trace Metals and Other Chemicals

Metals and metalloids can be harmful to human health through repeated exposure. They can cause damage to human health by promoting oxidative stress in cells (cadmium, chromium, lead, arsenic), neurological damage (lead, mercury), DNA damage (arsenic, chromium), and disruption of glucose (arsenic) or calcium (cadmium, lead) metabolism. Exposure to metals and metalloids can affect the immune, neurological, renal, endocrine, and reproductive systems, as well as placental functions. It is therefore of interest to study the effect of nutraceuticals on the toxicity induced by these compounds, as well as that induced by certain chemicals, and on neuroinflammation.

Chronic exposure to arsenic leads to neurotoxicity linked to intracellular ROS production. A model using PC12 cells and inorganic arsenic salt (iAs) was used to determine the ability of apigenin to counteract the effects of arsenic [114]. They were able to show that pre-treatment of cells with apigenin offered exceptional protection against iAs-induced neuroinflammation, but also against oxidative stress and cell death [114]. Nrf-2 appears to be involved in this neuroprotection [114].

Exposure to acrylonitrile, used in the production of synthetic fibers, rubber, and plastics, can cause pathological changes in the nervous system, which appear early after exposure and are very serious. Acrylonitrile induces oxidative damage and inflammation, which are implicated in its neurotoxicity. Apigenin can reduce oxidative stress, regulate the TLR4/NF-κB signaling pathway, and decrease interleukin-6 and TNF-α levels in acrylonitrile-treated rats [115]. Apigenin can also inhibit acrylonitrile-induced mitochondria-mediated apoptosis of neurons [115].

## 5. Conclusions

Neuroinflammation, implicated in various central nervous system disorders, underscores the importance of targeting inflammation pharmacologically. Apigenin, a flavonoid found in plant-based foods and beverages, has emerged as a potential therapeutic agent due to its anti-inflammatory properties.

Studies cited in this review have highlighted apigenin’s role in neuroinflammation across different pathologies: neurodegenerative diseases (multiple sclerosis, Parkinson’s disease, Alzheimer’s disease), cancer, cardiovascular diseases, cognitive and memory disorders, and toxicity related to trace metals and other chemicals. Evidence suggests that apigenin modulates various signaling pathways involved in inflammation, oxidative stress, and cell death, offering neuroprotective effects in experimental models. These common mechanisms include the NF-KB signaling pathway and the inhibition of NO synthase or COX2.

While promising, further research is needed to elucidate the precise molecular mechanisms underlying apigenin’s effects and evaluate its safety and efficacy in human populations. Despite these challenges, apigenin represents a promising avenue for the management of neuroinflammation-associated disorders.

In conclusion, apigenin holds potential as both a nutritional additive and complementary therapeutic agent, offering hope for improved management of neuroinflammatory conditions. Further investigations are warranted to translate preclinical findings into clinical applications effectively.

## Figures and Tables

**Table 1:** Mode of administration, dose, models, and mechanisms described for apigenin in multiple sclerosis.

Pathologies	Apigenin (Forms)	Dose	Models	Mechanisms	Ref.
Multiple sclerosis	Apigenin (Sigma Aldrich (St. Louis, MO, USA))	5, 10, and 20 µM	LPS-induced dendritic cells	Suppression of CD80, CD86, and major histocompatibility complex class I and II molecules, expressions on DCs Impairment of LPS-induced IL-12 expression	[80]
	Apigenin (Sigma Aldrich)	0, 1 and 25 µM	LPS-stimulated human monocytes, mouse macrophages	Inhibition in vivo of LPS-induced TNF and the mortality induced by lethal doses of LPS	[81]
	Reducing power of apigenin (according to the method of Oyaizu [87] and Amarowicz et al. [88]	0–20 µM	RAW 264.7 macrophage cells	Blocking NO-mediated COX-2 expression and monocyte adherence	[82]
	Apigenin (Sigma Aldrich)	10 µM	LPS-activated macrophages	Inhibition of inducible COX and inducible NO synthase promoter activities (action by PPAR)	[83]
	Apigenin solutions (supplier not specified)	1, 5, and 10 µM	BV-2 murine microglia cell line	Inhibition of the production of nitric oxide and prostaglandin E(2) by suppressing the expression of inducible NO synthase and COX-2 protein	[84]
	Apigenin (R&D System (Minneapolis, MN, USA))	40 mg/kg body weight	Experimental C57BL/6 autoimmune encephalomyelitis progression and relapse	Decrease expression of α4 integrin and CLEC12A on splenic DCs Increase in immune cell retention in the periphery compared to untreated EAE mice	[85]
	Apigenin (Sigma-Aldrich)	20 µM, 3 h	Dendritic cells isolated from PBMC	Shift of Th1- and Th17-type DC-modulated T-cell responses towards Treg-directed responses (decreased expression of T-bet, IFN-γ (Th1), and IL-17 (Th17) and increased expression of IL-10, TGF-β, and FoxP3 (Treg) in cells from normal human donors and EAE mice)	[86]

**Table 2:** Mode of administration, dose, models, and mechanisms described for apigenin in Parkinson’s disease.

Pathologies	Apigenin (Forms)	Dose	Models	Mechanisms	Ref.
Parkinson’s Disease	Apigenin (Sigma Aldrich)	20 mg/kg ip	Rat model of PD induced by rotenone	Attenuation of the upregulation of NF-κB gene expression. Prevention of the neuroinflammation in substantia nigra pars compacta. Inhibition of the release of pro-inflammatory cytokines TNF- α and IL-6 and pro-inflammatory enzyme iNOS-1.	[89]
	Apigenin (Chemscene (St. Louis, MO, USA))	Oral administration of AGN (25 and 50 mg/kg; p.o.) alone AGN (25 mg/kg; p.o.) in combination	LPS experimental model of rats	Decreased levels of nitrite, MDA, SOD, TNF-α, IL-1β, IL-6, and caspase-1. Increased levels of CAT, GSH, and complex-I. Modulation of NF-kB and Nrf2 signaling pathway.	[90]
	supplier not specified	50 mg/kg apigenin, 5 days	C57BL/6 mice treated by 1-methyl-4-phenyl-1,2,3,6-tetrahydropyridine	Reversion of the changes in expressions and concentrations of TNF-α, IL-1β, IL-6, IL-10, and TGF-β.	[91]

**Table 3:** Mode of administration, dose, models, and mechanisms described for apigenin in Alzheimer disease.

Pathologies	Apigenin (Forms)	Dose	Models	Mechanisms	Ref.
Alzheimer’s Disease	Isolated from the flowers of * Chrysanthemum boreale *	10 μM, 8 h	SH-SY5Y neuronal cells	Inhibition of caspase-3 activity.	[93]
	Apigenin (Sigma–Aldrich)	1 µM, 24 h	Glial cells and neurons were obtained from the brain hemispheres of Wistar rats	Reduce microglial activation Modulation of the mRNA expression of inflammatory cytokines, and reduced expression of OX42, IL-6, and gp130. Increase in the expression of brain-derived neurotrophic factor.	[94]
	Apigenin (Nutrafur S.A. (Alcantarilla, Spain))	20 mg/kg intraperitoneally	Male Wistar rats	Improve long-term memory.	[95]
	Apigenin (Sigma–Aldrich)	10, 20 and 40 mg/kg i.p	Male Wistar rats injected intraperitoneally with Streptozotocin	Decrease in MDA content, increase in SOD activity and GSH levels.	[96]
	Apigenin (Sigma–Aldrich)	25–100 mg/kg	22-month-old male Sprague Dawley rats	Restore histone acetylation and BDNF signaling. Suppression of isoflurane exposure induced upregulation of pro-inflammatory cytokines and NFκB signaling pathway.	[97]
	Huike Botanical Development Company (Xi’an, China)	oral gavage 5 days/week at a dose of 40 mg/kg body weight once a day	APP/PS1 double transgenic mice	Restore neurotrophic ERK/CREB/BDNF pathway in the cerebral cortex. Down-regulation of BACE1 and β-CTF levels, the relief of Aβ deposition. Decrease in insoluble Aβ levels	[98]
	Apigenin (Nutrafur S.A.)	110 mg/kg per day	C57BL/6 and GFAP-IL6 heterozygous mice	Decrease in the number of Iba-1+ microglia in the hippocampus of GFAP-IL6 mice and change in microglial morphology.	[102]
	Apigenin (Sigma-Aldrich)	X	Molecular docking	Binding of apigenin with human transferrin. Stability of human transferrin–apigenin complex. Targeting neuroinflammation by apigenin in the context of iron homeostasis.	[103]
	Apigenin contained in *B. pendula* leaf extract	200 mg/kg b.w. of * B. pendula * leaf extract	Wistar rats with intracerebroventricular injection of Aβ_1-42_	Decrease in inflammation (cytokines: tumor necrosis factor-α (TNF-α), Interleukin 1β (IL-1β), and cyclooxygenase-2 (COX 2)) in plasma and hippocampus homogenates.	[104]
	Apigenin (Sigma-Aldrich)	25 mg/kg	Zebrafish treated with aluminum	Neutralization increased expression of genes related to neuroinflammation.	[105]

**Table 4:** Mode of administration, dose, models, and mechanisms described for apigenin in cancer and cardiovascular diseases.

Pathologies	Apigenin (Forms)	Dose	Models	Mechanisms	Ref.
Cancer	Combination of PMA and apigenin (Sigma Aldrich)	30 µM of the tested molecules	Human brain microvascular endothelial cells	Inhibition of MMP-9 increase induced by PMA. Reduction in BBB disruption via inhibition of NF-κB signaling pathway.	[107]
Cardiovascular disease	Apigenin solutions (supplier not specified)	1, 5, and 10 µM	Primary microglia (1-day-old Spraque-Dawley rats) + LPS	Reduction in NO production and iNOS protein levels in dose-dependent manners.	[84]
			BV-2 cells (murine microglial cells) + LPS	Reduction in NO production and iNOS protein levels in dose-dependent manners. Inhibition of PGE2 caused by reduction in COX-2 expression. Slight reduction in COX-1 expression. Inhibition of JNK and p38 MAPK phosphorylation.	
	Apigenin solutions (supplier not specified)	Oral administration (20 mg/kg) 30 min after MCAO	Adult male ICR mice with MCAO—sacrifice 22, 5 h after reperfusion	Reduction in the infarct volume. Decrease in the number of OX-42-positive cells.	[84]
	Flavonoid-rich extract (FRE) from *Rosa laevigata* Michx. fruit (Yunnan Qiancaoyuan Pharmaceutical Company Co., Ltd. (Yunnan, China))	50, 100, and 200 mg/kg of FRE	Male Sprague Dawley rats with MCAO	Decrease in the expressions of NF-κB, iNOS, MMP-9, COX-2, TNF-α, IL-1β, IL-4, and IL-6. Down-regulated the levels of p-JNK, p-ERK, and p-p38 in MAPK pathways. Anti-inflammatory properties of FRE.	[108]
	VO Ex	200, 400, 800 mg/kg with 0.22 ± 0.01 mg/g of VO Ex	Adult male Sprague Dawley rats with MCAO	Reduction in the infarct volume, structural damage, and neuronal death. Inhibition of IL17A, IL1β, MMP9 and MMP3 mRNA levels. Decrease in IL-7, IL-6, and TNF-α protein expression.	[109]
	Apigenin (Baoji Chenguang Biotechnology Co., Ltd. (Baoji, China))	6.25, 12.5, 25, 50, and 100 μM for 4 h before OGD/R	Primary rat astrocytes for cell viability assay or with OGD/R	Improvement of the cell viability with the OGD/R injury group.	
	VALAH	Verbenalin (75 μM), acteoside (50 μM), luteolin (6.25 μM), apigenin (6.25 μM), and hispidulin (12.5 μM)	Primary rat astrocytes for cell viability assay or with OGD/R	Increase the cell survival rate in a dose-dependent manner. Inhibition of IL17A upregulation. Decrease in IL1-β, IL6, MMP9, and TNF-α protein expression levels.	

PMA: phorbol 12-myristate 13-acetate; LPS: Lipopolysaccharide; MCAO: middle cerebral artery occlusion; PGE_2_: prostaglandin E_2_; OGD/R: oxygen and glucose deprivation/reoxygenation; VO Ex: extract of Verbena officinalis; VALAH: verbenalin (75 μM), acteoside (50 μM), luteolin (6.25 μM), apigenin (6.25 μM), and hispidulin (12.5 μM).

**Table 5:** Mode of administration, dose, models, and mechanisms described for apigenin in cognitive and memory disorders, and toxicity related to trace metals and other chemicals.

Pathologies	Apigenin (Forms)	Dose	Models	Mechanisms	Ref.
Cognitive and memory disorders	Apigenin (Sigma Aldrich)	25, 50, and 100 mg/kg	22-month-old male Sprague Dawley rats treated with isoflurane	Reduction in isoflurane induced neuroinflammation, restoring IL-2, IL-4, and IL-10 levels via modulation of the NF-κB inflammatory pathway	[97]
	Hydroalcoholic extract from *M. chamomilla* dry flowers (Apigenin-7-glucoside 927.62 mg/100 g dry flowers and Apigenin 377.64 mg/100 g dry flowers)	25 or 75 mg/kg of hydroalcoholic extract	Male Wistar/25 rats (4 months old) treated with scopolamine	Reversal of decreased BDNF mRNA levels and increased IL1β mRNA levels in hippocampal tissue after scopolamine treatment	[111]
	(Poly)phenol content of foods from the Phenol-Explorer database.		Adults living in southern Italy	Consumption of certain (poly)phenols associated with better sleep quality, possible involvement of neuroinflammation	[112]
	Apigenin (Fisher Scientific)	0.5 mg/mL, in 0.2% carboxymethylcellulose, in drinking water for 6 weeks	Young and old C57BL/6N mice	Modulation of transcriptomic signatures of inflammation/immune activation Reversal of gene expression signatures related to inflammation and immune activation in the aging mouse brain	[113]
		25 μM for 24 h	Primary human astrocytes	Reduced markers of senescence and inflammation in aging-like primary human astrocytes	
Toxicity related to trace metals and other chemicals	Apigenin (Sigma Aldrich)	0.01–300 μM for 24 or 48 h	PC12 cell line derived from a pheochromocytoma of the rat adrenal medulla	Protection against iAs-induced neuroinflammation, oxidative stress, and apoptotic cell death via Nrf2 upregulation	[114]
	Apigenin (Shaanxi Ci Yuan Biotechnology Co., Ltd. (Xi’an, China))	117, 234, and 351 mg/kg	Rats treated with acetonitrile	Reduced oxidative stress, down-regulated the TLR4/NF-κB signaling pathway, decreased the levels of IL-6 and TNF-α, and inhibited mitochondria-mediated neuron apoptosis	[115]
